# Statement of Retraction: LncRNA SOX2-OTinhibitionprotects against myocardialischemia/reperfusion-inducedinjury via themicroRNA-186-5p (miR-186-5p)/Yin Yang 1 (YY1)pathway

**DOI:** 10.1080/21655979.2024.2299620

**Published:** 2024-02-20

We, the Editors and Publisher of the journal Bioengineered, have retracted the following article:

Pengjie Yang, Kun Liang, Weisong Wang, Dehua Zhou, Yuan Chen, Xueyan Jiang, Rong Fu, Benben Zhu & Xuefeng Lin (2022) LncRNA SOX2-OTinhibitionprotects against myocardialischemia/reperfusion-inducedinjury via themicroRNA-186-5p (miR-186-5p)/Yin Yang 1 (YY1)pathway, Bioengineered, 13:1, 280-290, DOI: 10.1080/21655979.2021.2000229

Since publication, significant concerns have been raised about the integrity of the data and reported results in the article. When approached for an explanation, the authors did not provide their original data or any necessary supporting information. As verifying the validity of published work is core to the integrity of the scholarly record, we are therefore retracting the article. All authors listed in this publication have been informed.

We have been informed in our decision-making by our editorial policies and the COPE guidelines.

The retracted article will remain online to maintain the scholarly record, but it will be digitally watermarked on each page as ‘Retracted’.

